# Can Non-Phase-Transformation Heat Treatments Improve the Strength Properties of Materials?

**DOI:** 10.3390/ma18071599

**Published:** 2025-04-01

**Authors:** Adrian Neacșa, Ibrahim Naim Ramadan, Alin Diniță, Ștefan Virgil Iacob, Costin Nicolae Ilincă, Eugen Victor Laudacescu

**Affiliations:** 1 Department of Mechanical Engineering, Petroleum-Gas University of Ploiesti, 100680 Ploiesti, Romania; adnea@upg-ploiesti.ro (A.N.); icostin@upg-ploiesti.ro (C.N.I.); leugen@upg-ploiesti.ro (E.V.L.); 2 Industrial Engineering and Robotics Faculty, Politehnica University of Bucharest, Spl. Independentei 303, 060042 Bucharest, Romania; 3 Economic Sciences Faculty, Petroleum-Gas University of Ploiesti, 100680 Ploiesti, Romania

**Keywords:** physical and mechanical characteristics, steel, non-phase-transformation heat treatments, mechanical engineering, manufacturing applications

## Abstract

The article is the result of the question mentioned in its title, namely, whether heat treatments without phase transformation and pressing of parts can improve the physicomechanical properties of metallic materials and alloys. Starting from this hypothesis, the article analyzes the influence of non-phase change thermal treatment TT and plastic deformation (compression) on a steel used for the realization of components in the engineering industry, as presented in the specific standards SR EN-10025 and SR EN-10027. The results of the tensile tests and of the Vickers hardness tests on the specimens made of this material are presented. The results in terms of material ultimate stress σ_u_, yield strength S_y_, elongation δ, reduction in cross section ψ, as well as those obtained in the Vickers test are summarized in tabular or graphical form. From the research conducted by the authors of this work, it can be seen that the 0.5 × T_m−s_ (T_m−s_—melting-solidifying temperature, K) heat treatment gives the best mix of properties: mechanical strength similar to that of the non-treated material, improved elasticity and ductility, but with a small, negligible reduction in hardness. The results are useful to support the activities of optimal selection of heat treatments and plastic forming for various engineering applications. Heat treatment without phase transformation is essential for improving the mechanical properties of materials used in engineering. This study investigates the impact of heat treatments and plastic deformation on S355J2+N steel, highlighting the increase in yield strength and improvement in ductility. The results show an increase of up to 15% in yield strength and an improvement in relative elongation by 2% for treatments at 0.5 × T_m−s_, while hardness remains almost unchanged.

## 1. Introduction

Heat treatments are fundamental in optimizing the performance of materials used in engineering. Although phase transformations are well studied, non-phase transformation treatments offer considerable potential for improving mechanical properties. The present study focuses on the effects of non-phase heat treatments on steel components.

Annealing of metallic materials and alloys does not involve phase transformations, but is intended to correct deviations from equilibrium caused by technological processes of casting, plastic deformation, welding, heat treatments or other previous technological processes (segregation, strain hardening, internal stresses, etc.), used for homogenization of chemical composition, recrystallization and stress relieving, by controlled heating to certain temperatures and appropriate holding times [1,2,3,4,5].

Thus, the basic parameters of the treatment parameters are the heating temperature and the holding time, while the heating and cooling conditions are of secondary importance [6,7].

The technological processes have an effect on the structure of the polycrystalline aggregates and produce the following effects:

➢ Shape and size of crystalline grains change [8,9]. The grains rotate to adopt favorable positions with respect to external stresses, elongate in the direction of deformation, forming a fibrous structure. Brittle phases and inclusions break up and align in the same direction.
➢ Changes in the crystalline lattice orientation [10,11,12]. Before plastic deformation, the crystallographic planes exhibit different orientations, varying by tens of degrees between grains, with the crystal lattice aligning in a preferred direction, thus producing a deformation-specific texture.
➢ Changing the internal structure of each grain [13,14]. During plastic deformation, 10–20% of the mechanical energy used is stored in the lattice in the form of defects in the crystalline structure, which is why zones with different crystallographic orientations (mosaic structure) appear inside the grains, delimited by marginal dislocations, substructures, point or packing defects.

Non-phase transformation is a phenomenon that affects metallic materials, including carbon steels. Also, non-phase transformation occurs when changes in microstructure are induced by certain temperature and deformation conditions, without the material undergoing a distinct phase or a clearly defined phase change. These transitions can significantly influence the mechanical properties of steels, particularly in strength and hardness. Below are some mechanistic explanations for why non-phase transformation can improve the strength properties of carbon steels.

Dislocations are defects in the crystal lattice of a material that move under the action of mechanical stress. In carbon steels, the presence and behavior of dislocations play an important role in the resistance to plastic deformation. Non-phasic transitions induce the formation of additional dislocations and influence the way the dislocations move, which leads to improved material strength. Non-phase transformations can also result in the completion of grains, thereby enhancing the resistance of the material to dislocation movement through the ˝Halling˝ hardening mechanism. During non-phase transitions, the presence of interstitial atoms hinders the mobility of dislocations, thereby facilitating a hardening mechanism.

The strength of steels is significantly impacted by residual stress, which persists in the material even after external forces are removed. During non-phase transformations, favorable residual stresses can develop, enhancing resistance to plastic deformation and fatigue. These residual stresses frequently result from interactions between crystal lattice atoms and dislocations. Some phase change-free heat treatments induce beneficial residual stresses, thereby enhancing the material’s resistance to plastic deformation. By controlling the deformation process (e.g., rolling or forging), residual stress distributions can be engineered to promote material hardening.

The grain size of steels is a critical factor in their mechanical behavior. A reduction in grain size results in enhanced material strength due to the barrier effect on dislocation movement. Heat treatments that do not involve phase change can lead to a finer-grained microstructure, characterized by this refinement has been shown to enhance the strength of the material by restricting dislocation movement and impeding crack propagation, thereby promoting improved fracture and fatigue strength. Under specific conditions, the absence of phase transitions can lead to rapid recrystallization, a process that significantly reduces grain size and consequently strengthens the material.

Heat treatments devoid of phase change can give rise to the formation of precipitates within the metal matrix or solid solutions, which in turn exert influence on the strength properties of the material. The presence of fine precipitates or solid solutions during the heat treatment process can impede dislocation movement, thereby inducing a hardening effect on the material. In the case of carbon steels, heat treatments devoid of phase change result in the formation of solid solutions, which can enhance material strength through solid solution hardening mechanisms.

These changes lead to work hardening of the material, which means increased strength properties and reduced plasticity. The resultant texture and fibrous structure give rise to anisotropic properties, with particular effects on toughness and elongation at break.

The resulting structure has a high energy level which leads to a tendency to transform by diffusion-based processes to reach the steady state. Depending on the heating temperature of the cold deformed material, these transformations include recovery, primary crystallization, grain growth and secondary crystallization.

## 2. Literature Review

A review of the existing literature highlights the advantages of non-phase transformation heat treatments for steel. In contrast to other studies, this research explores the effects of subcritical temperature treatments on S355J2+N steel (S355J2+N is a variant of S355J2 that includes the “+N”, indicating it has undergone additional processing through controlled rolling or thermo-mechanical treatment after normalizing, resulting in improved properties.).

Annealing without phase change is a heat treatment process that is used to modify the properties of metallic materials without changing their crystalline structure. For the purpose of this article, the main types of annealing, together with their objectives and relevant bibliographic coordinates, are presented below.

➢ Restoration

The studies carried out on this heat treatment [15,16] underline the fact that, when this non-phase change heat treatment is applied, transformations occur which are only visible by X-ray or electron microscope analysis and which are materialized by the reduction of dislocations, the attenuation of internal stresses and the recovery of ductility, without affecting the crystalline structure. The temperature range (relation 1) is below the recrystallization temperature (typically 200–400 °C for metallic materials).

(1) $$T_{c}=\left(0.2\ldots 0.3\right)\times T_{m\text{−}s}$$

where: T_c_—temperature range of the restoration heat treatment;

T_m−s_—melting-solidifying temperature range, K.

➢ Primary recrystallization

The effect of this heat treatment on the recrystallization of the alloys is shown in the studies presented in the literature [17,18]. Complete recrystallization occurs after 30 min of annealing at 620–700 °C, producing equiaxial crystals. The activation energy for recrystallization is quantified and the process is related to improved mechanical properties such as tensile strength and elongation. These studies also focus on recrystallization mechanisms, microstructure optimization by heat treatments, and the interaction between temperature, time, and deformation parameters. Research shows how processing techniques, such as shot peening and subsequent heat treatments, influence recrystallization in alloys. Recrystallization conditions are evaluated to optimize properties for high performance applications such as aerospace components.

The analysis of other literature [19,20] show that this type of heat treatment is carried out for partial modification of the structure of materials, especially in the case of multiphase alloys, without homogenization of the structure. In the temperature range (Relation 2) slightly below the phase transformation threshold specific to the alloy system, a structure consisting of equiaxially unmixed grains is formed.

(2) $$T_{c}=\left(0.3\ldots 0.4\right)\times T_{m\text{−}s}$$

where: T_c_—temperature range of the primary recrystallization heat treatment;

T_m−s_—melting-solidifying temperature range, K.

➢ Secondary recrystallization

Secondary recrystallization is a thermal process that occurs after primary recrystallization of a material, usually to modify its structure and properties, such as to produce a material with improved strength or durability. This process is commonly used to process metallic materials such as steel or aluminum, but also used in other fields such as ceramics. The metallurgical or materials literature contains detailed sections on secondary recrystallization, such as studies of the influence of temperature and composition on the process, investigations of crystal growth mechanisms, or analysis of structural changes after recrystallization. It is emphasized in the literature [21,22,23,24,25,26,27,28] that during secondary recrystallization the structure of the material is modified by growing large crystals which occur after primary recrystallization (when small crystals and dislocations in the material are removed) ends. This process usually results in a reduction in internal stress and an improvement in mechanical properties, and takes place at higher temperatures than primary recrystallization.

➢ Stress relief annealing

This non-phase change heat treatment is used to reduce residual stresses induced by cold working, pre-machining or welding without significant microstructural changes, as shown in the studies in the two bibliographic works [29,30]. Stress relieving can also be carried out as a secondary process to other treatments, provided that heating and cooling are carried out at low rates to avoid thermal stresses and to ensure transformations under steady-state conditions, and the temperature range usually lies between 100 and 300 °C, depending on the type of metal or alloyed material. Of course, over time, a series of heat treatment methods and techniques have been experimented and applied, such as electrically assisted annealing, which has the advantage of reducing process time by superimposing the effect of electroplasticity on that of high temperature or double annealing which is more effective in terms of crack expansion in the material by reducing stress at different stages of thermal fatigue tests [31,32,33].

➢ Subcritical annealing (low-temperature annealing)

It has been shown in the literature [34,35] that the application of this type of heat treatment results in a reduction in the brittleness of steel, usually after hardening (tempering heat treatment is related but distinct). For steels, a temperature range of 150–300 °C is recommended.

**Figure 1:**
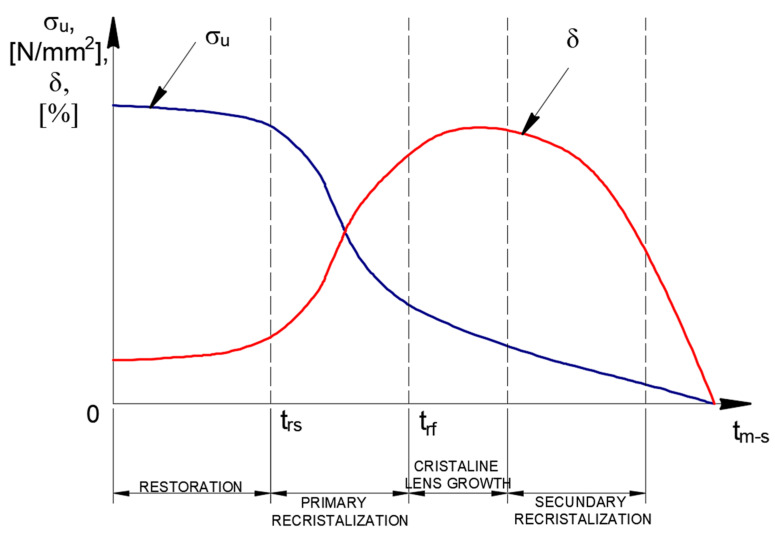
Variation of mechanical characteristics after recrystallization annealing without phase change. Source: Authors based on the content of the literature review [7,8,10,11,12,13,14].

**Figure 2:**
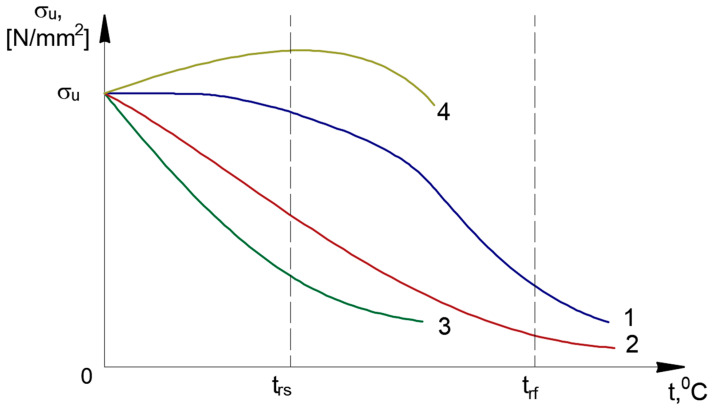
Ultimate tensile strength after recrystallization annealing without phase change—special cases. 1. Cu, Ag, Ni; 2. Fe, Al; 3. W, Mo; 4. High yield strength alloys of Cu, Ni for springs and membranes. Source: Authors based on the content of the literature review [7,8,10,11,12,13,14].

Synthesizing the data obtained from the biliographic analysis, it appears that the evolution of the mechanical properties after applying the recrystallization annealing treatment without phase change follows the suggestive trends shown in Figure 1 and Figure 2.

Some general observations can also be made:

- Heat treatments below 727 °C are mainly aimed at microstructure refinement, stress relief and phase transformations, avoiding complete recrystallization.
- The types of metallic materials or alloys, heating times and cooling methods have a significant influence on the results.

While maintaining a balance between ductility and toughness, studies consistently show an improvement in mechanical properties such as strength, fatigue and wear resistance.

Faster cooling rates after intercritical annealing increase heterogeneity in carbide morphology, which may require post-treatment annealing.

Cross-industry applications can be found in bearings (SAE 52100), springs (EN47) and cutting tools (H13 tool steels). All benefit from these treatments, demonstrating their adaptability and versatility across industries.

The ensuing case study demonstrates how precise temperature control below 727 °C and suitable holding times can substantially enhance the strength and toughness of S355J2+N steel.

## 3. Materials and Methods

The heat treatment temperatures (0.2 × T_m−s,_ 0.4 × T_m−s,_ 0.5 × T_m−s_) were selected based on the findings of prior studies on the optimization of mechanical properties. The equipment used was calibrated to minimize measurement errors, and the tests were repeated to ensure reproducibility of the results, in order to minimize errors.

**Figure 3:**
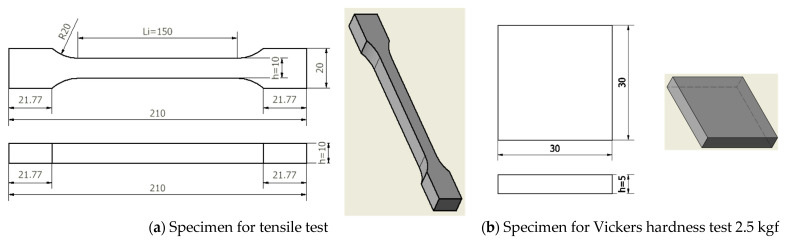
Design of the standardized specimens and for: (**a**) tensile test; (**b**) Vickers hardness test 2.5 kgf. Source: Authors based on the content of tensile and hardeness test standards mentioned above.

The study proposed a test program for 60 specimens of steel S355J2+N, consisting of 30 specimens for tensile test and 30 specimens for Vickers hardness test 2.5 kgf (Figure 3).

According to the regulations of SR EN-10025 [36] and SR EN-10027 [37], the steel subjected to this experimental program, symbolized by S355J2+N, is a structural steel with yield strength of 355 N/mm^2^ and an energy limit of 27 J at −20 °C, guaranteed by the manufacturer. It is thermomechanically rolled.

The mechanical properties are guaranteed by the quality certificates, and the chemical composition is that resulting from the analysis certificates when the steel shims from which the specimens were taken were prepared, as shown in Table 1 and Table 2.

From the material under test, S355J2+N, 60 standardized and numbered specimens were made, as shown in Table 3 and Table 4.

The specimens were exposed to annealing heat treatments devoid of phase change at the temperature levels also delineated in Table 3 and Table 4. They were then maintained within a thermostated high-temperature furnace Snol of Lithuanian provenance (Snol, Umega Group, AB, SnolTherm Dpt., Narcűnai, LT-28104, Utena, Lithuania) for a duration of 24 h. Thereafter, a cooling process was initiated following the furnace’s cooling down. Also, a force of 1000 kN was utilized to fabricate the four sets of specimens for tensile and hardness tests, which were compressed with 1% and 2% of their maximum capacity.

The tests for the determination of the physical-mechanical characteristics were carried out in accordance with EN ISO 6892-1:2019 Metallic materials—Tensile testing—Part 1: Method of test at room temperature [38].

The first part of this standard details the test method to be employed at room temperature, utilizing a universal static and dynamic testing machine with a capacity of 300 kN. This machine, designated as the Walter + Bai LFV, was manufactured by Walter + Bai AG Prüfmaschinen—Testing Machines, a company based in Löhningen, Switzerland.

Tests were conducted to ascertain the hardness characteristics of the subject material. These tests were carried out in accordance with EN ISO 6507-1:2023 Metallic materials—Vickers hardness test—Part 1: Test method, which is the standard for the Vickers hardness test of metallic materials [39].

The test method is delineated in Part 1 of the aforementioned standard. The Emcotest DuraScan 20, manufactured in Wiehl-Marienhagen, Germany, was utilized as the testing machine.

The following is a summary of the data recorded from the tensile and Vickers hardness test in accordance with EN ISO 6892-1:2019 Metallic materials—Tensile testing—Part 1: Method of test at room temperature and EN ISO 6507-1:2023 Metallic materials—Vickers hardness test—Part 1: Test method, for all six categories of standardized specimens in Table 3 and Table 4. All the test conditions were observed during the tests and the specimen dimensions are those specified in the above-mentioned standard, and the data obtained are shown in Table 5 and Table 6.

A statistical analysis was conducted to interpret the reliability of the outcomes derived from the tensile tests (ultimate material strength, yield strength, elongation) and the Vickers hardness test (2.5 kgf). The analysis revealed no substantial deviations from the calculated mean for each sample set. This finding is corroborated by the calculated values of standard deviation, which confirm the precision of the test results. Consequently, the test results for each sample set exhibited a high degree of homogeneity, thereby confirming the successful execution of the tests and validating the obtained results.

**Figure 4:**
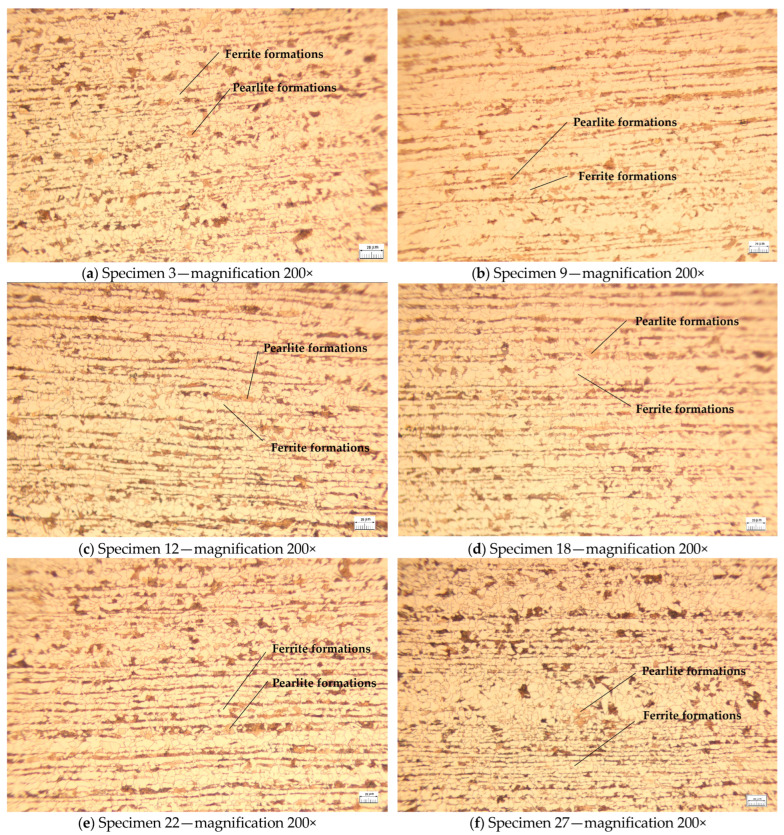
The microstructures of ferrites and perlites in the specimen samples were examined using an optical metallographic microscope: (**a**) without heat treatment or plastic deformation; (**b**) with heat treatment without phase change at 0.2 × T_m−s_; (**c**) with heat treatment without phase change at 0.4 × T_m−s_; (**d**) with heat treatment without phase change at 0.5 × T_m−s_ (**e**) without heat treatment under 1% compression; (**f**) without heat treatment under 2% compression.

**Figure 5:**
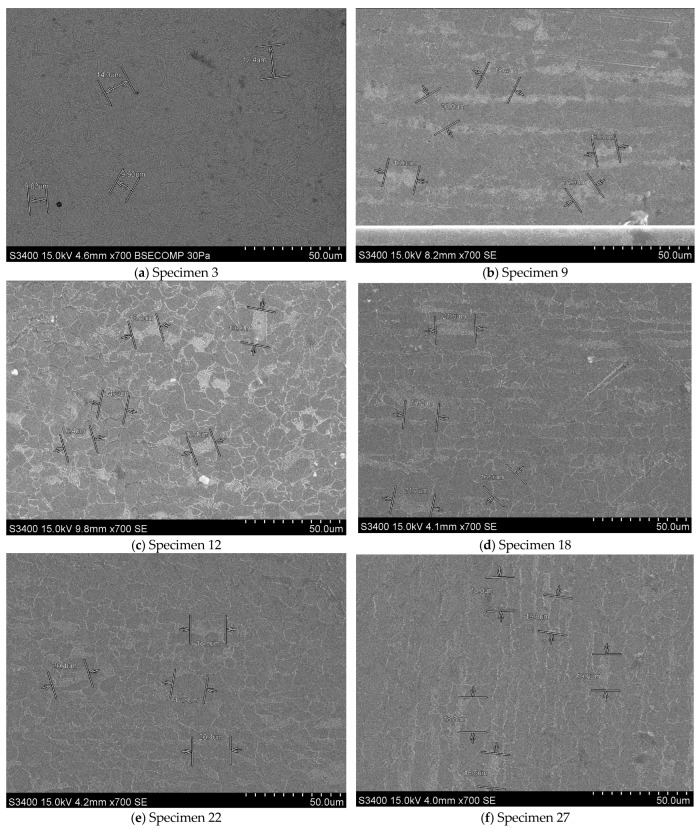
Ferrite and perlite microstructures of specimens analyzed by scanning electron microscope (SEM): (**a**) without heat treatment or plastic deformation; (**b**) with heat treatment without phase change at 0.2 × T_m−s_; (**c**) with heat treatment without phase change at 0.4 × T_m−s_; (**d**) with heat treatment without phase change at 0.5 × T_m−s_ (**e**) without heat treatment under 1% compression; (**f**) without heat treatment under 2% compression.

The microstructures of a single specimen randomly selected from each set of specimens subjected to both tensile and Vickers 2.5 kgf hardness tests are shown in Figure 4 and Figure 5. The metallographic analysis of the specimens was conducted using a metallographic optical microscope manufactured in Japan by Olympus. Specifically, the BX60M Reflected Light Brightfield & Darkfield Microscope (Hachioji, Tokyo, Japan) was employed, along with a Hitachi S-3400N Scanning Electron Microscope (Marunouchi, Chiyoda, Tokyo, Japan).

The specimens were examined to ascertain the effects of heat treatment and plastic deformation. The specimens without these treatments show a homogeneous microstructure, retaining the characteristics of the base material (initial phase), higher grain size, and less dense structure. The microstructure of the specimens revealed a clear distribution of pearlitic and ferritic formations, indicating a lack of thermal modification processes. The intercrystalline zones between the ferrite and the pearlite were well delimited, confirming that the specimens retained a structure characteristic of the material in its initial state.

Specimens 9, 12, and 18 underwent heat treatment without phase transformation at varying relative melting point temperatures (T_m−s_, K). The observed differences indicate that Specimens 9 exhibits a less modified grain size compared to Specimens 12 and 18. In these specimens, the material structure is more susceptible to thermal diffusion. Additionally, a slight increase in microstructural homogeneity is observed for Specimen 9 compared to Specimen 3. The crystal boundaries highlight a partial relaxation and the pearlitic structure becomes more uniform as coalescence initiates in the granular micro-structure, albeit without significant alterations.

In Specimen 12, a substantial augmentation in the dimensions of the ferrite and pearlite formations is discernible. The onset of thermal diffusion engenders a flattening of the structures, accompanied by the diffusion of boundaries and a decline in material hardness at this temperature.

In contrast, Specimen 18 exhibited a notable reduction in the visibility of intercrystalline boundaries between the ferritic and pearlitic phases, accompanied by an increase in the size and homogeneity of the crystalline grains. This observation suggests a structure that is susceptible to imminent recrystallization.

As the heat treatment temperature is increased, without undergoing phase change, the grain sizes may become larger and their boundaries more diffuse.

In the cases of Specimens 22 and 27, the specimens were not subjected to heat treatment, however, they were exposed to 1000 kN of mechanical compressive load. In Specimen 22, initial indications of plastic deformation manifested as dislocations and the alignment of the intercrystalline boundaries in the direction of the applied force. The specimen reveals a microstructure characterized by minor dislocations and local elastic deformations. The structure is more chaotic than in the heat-treated specimens, and the defect density is moderate. For Specimen 27, a microstructure with a higher density of dislocations and deformed plastic zones with a more pronounced deformation of the intercrystalline boundaries is observed. The targeting of the structures depending on the applied load is evident. A comparison of this specimens to Specimen 22 exposes that the material undergoes more severe plastic deformation, manifesting in a more distorted intercrystalline boundary arrangement.

The aforementioned observations indicate that the heat treatment promotes grain growth and eliminates defects. Conversely, the application of mechanical loads induces dislocations and deformations within the crystalline microstructure.

As demonstrated above, the application of heat treatment promotes grain growth and eliminates defects. Conversely, the application of mechanical loads induces dislocations and deformations within the crystalline microstructure.

In contrast to the heat-treated specimens, Specimens 22 and 27 have more chaotic microstructures and higher defect densities, as evidenced by microscopic analysis.

It is evident that the heat treatment promotes grain growth and the relaxation of crystalline boundaries, leading to a more homogeneous structure. In contrast, the application of mechanical loads without prior heat treatment induces substantial crystalline dislocations and deformations, as evidenced by the density and directionality of the structures. The specimens that underwent both heat treatment and mechanical forces exhibited enhanced properties in terms of hardness and ductility, suggesting a potential follow-up variant of this study.

By integrating the outcomes of mechanical tests with the structures depicted in Figure 4 in terms of mechanical characteristics, the following correlations can be discerned.

The specimens that underwent neither heat treatment nor mechanical loading (Specimen 3) exhibited standard strength, as guaranteed by the manufacturer, and a hardness char-acteristic of the raw material. However, these specimens demonstrated limited toughness and ductility due to the absence of heat treatment, which is necessary to optimize the structure. It is therefore concluded that the material exhibits a susceptibility to stress concentration in the region of the intercrystalline boundaries.

It is observed that Specimen 9 has a structure that begins to homogenize, and the crystal boundaries are partially relaxed, causing a slight increase in ductility without a significant decrease in strength.

In contrast, Specimen 12 exhibited an augmentation in the granular size of the pearlitic and ferritic phases, signifying an enhancement in ductility and malleability. However, this transition concomitantly entailed a diminution in initial hardness and an elevated propensity for plastic deformation.

In contrast, Specimen 18 exhibited a diffuse crystalline structure, indicating a significant reduction in crystallinity. This pecimen demonstrated a softening and a loss of strength relative to the other specimens. Consequently, the material’s potential for applications requiring high resistance to mechanical stresses is limited.

The unheat-treated specimens subjected to compression exhibit mechanical character-istics of the hardened structures by roughening. Thus, in Specimen 22, early dislocations occur, but the material maintains and exhibits good strength. The slight increase in mechanical strength is due to the strain-hardening phenomenon. It shows limitations at higher deformations without heat treatment.

For Specimen 27, a high dislocation density and obvious plastic deformation is observed. The material exhibits significant hardening, but is more susceptible to embrittle-ment leading to an increased risk of cracking under repeated loads. Ductility is reduced to a level comparable to that of Specimen 22.

Electron microscopy (SEM) images at 700× magnification illustrate the microstructure of S355J2+N steel specimens under different heat treatment and plastic deformation conditions (Figure 5). The crystallographic ferritic-pearlitic microstructure, along with the alterations induced by the applied treatments and 1% and 2% compression, can be readily discerned. As was the case in the metallographic optical microscope analysis, a concise commentary is hereby presented, drawing upon the findings from the microstructure analysis.

For specimen 3, which belongs to the category of specimens not subjected to heat treatment or plastic deformation, a uniform microstructure is observed. This microstructure is characteristic of the analyzed steel and is unaffected by plastic treatments or deformations. The grain size in this specimen is relatively homogeneous, and the distribution of phases (ferrite and pearlite) exhibits minimal discontinuities.

In specimen 9, which underwent heat treatment without phase change at 0.2 × T_m−s_, a slight refinement of the microstructure is observed, accompanied by a tendency toward clearer organization of the crystalline graunites. The pearlitic phase is also well delineated; however, there are no significant changes in the phase ratio.

In specimen 12, which underwent a heat treatment without phase change at 0.4 × T_m−s_, a finer microstructure is evident compared to specimen 9. The differentiation between the two phases, the ferritic and the pearlitic, is more pronounced, suggesting an influence of the heat treatment on carbon diffusion.

For specimen 18, which underwent a heat treatment without phase change at 0.5 × T_m−s_, the microstructure exhibited a distinct tendency towards refinement of the crystalline grains of ferrite and perlite. Furthermore, the specimen manifests more regular shapes and a more uniform phase distribution, indicative of an emerging structural rearrangement.

In contrast, specimen 22, which underwent no heat treatment and was compressed at 1%, exhibited deformation in its microstructure, with a predominant orientation of the grains aligning with the direction of compression. The plastic deformation influences the distribution of the phases, resulting in elongated contours along the direction of compression.

In the case of specimen 27, which underwent no heat treatment and was compressed with 2%, the deformation effects are more pronounced, exhibiting a discernible orientation of ferritic-pearlitic structures along the deformation direction. Consequently, the crystalline grains become more elongated, and lines of deformation become evident, indicating that the structure has been subjected to substantial stress.

Heat treatments applied below the critical temperature A_c1_ did not produce major phase changes; however, they influenced the microstructure distribution and refinement. The application of plastic deformation led to grain elongation and the occurrence of dislocations typical of compression processes. The steel S355J2+N demonstrated a stable ferritic-pearlitic microstructure, indicating potential for enhancement of its mechanical properties through the implementation of heat treatment and controlled deformation.

From the point of view of the applied heat treatments, a relaxation of the intercrystalline boundaries is evidenced, reducing the level of internal stresses and increasing the ductility of the material, but this may decrease the mechanical strength. Higher temperatures favour recrystallization, resulting in a softer but more uniform structure.

In terms of mechanical deformation, the application of compression without heat treatment leads to dislocation hardening, which leads to an increase in hardness and a reduction in ductility. Specimens subjected to compression (in particular Specimens 27) become more susceptible to cracking and consequently to brittle failure. The results of the experimental programs will be presented, some conclusions will be drawn and some discussions will be initiated on the basis of what has been highlighted from the point of view of the materials and test methods used in the following subchapters.

## 4. Results

**Figure 6:**
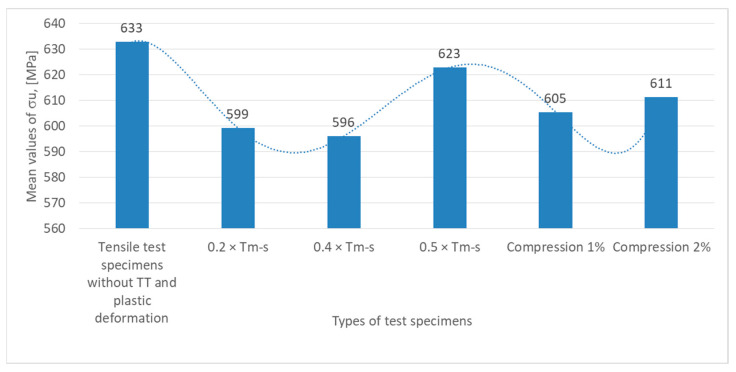
The mean values of the material strength recorded in the tensile test for the six types of test specimens are as follows.

**Figure 7:**
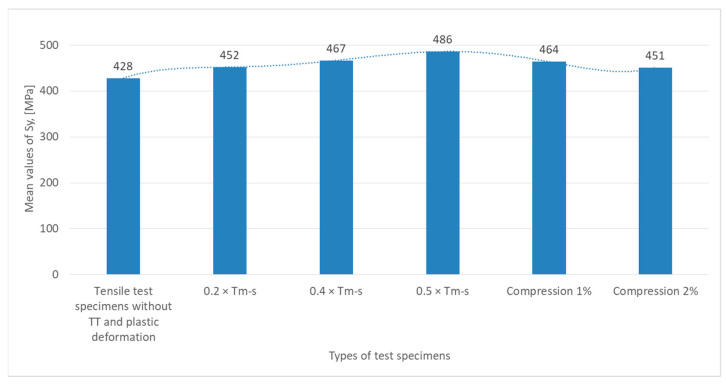
The mean values of the yield strength S_y_, corresponding to a specific elongation ε = 0.002, recorded in the tensile test for the six types of specimens tested.

**Figure 8:**
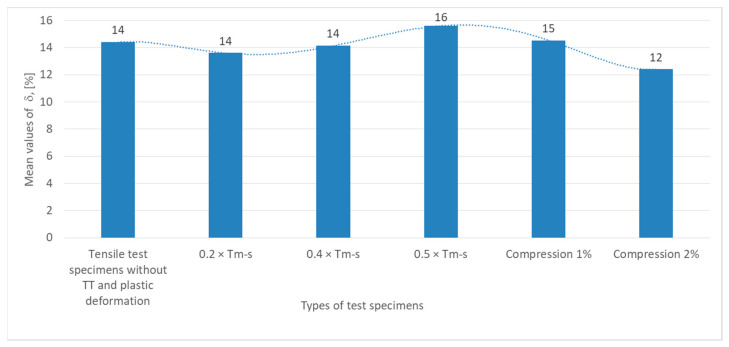
The mean values of the relative material elongations recorded in the tensile test for the six types of specimens tested.

**Figure 9:**
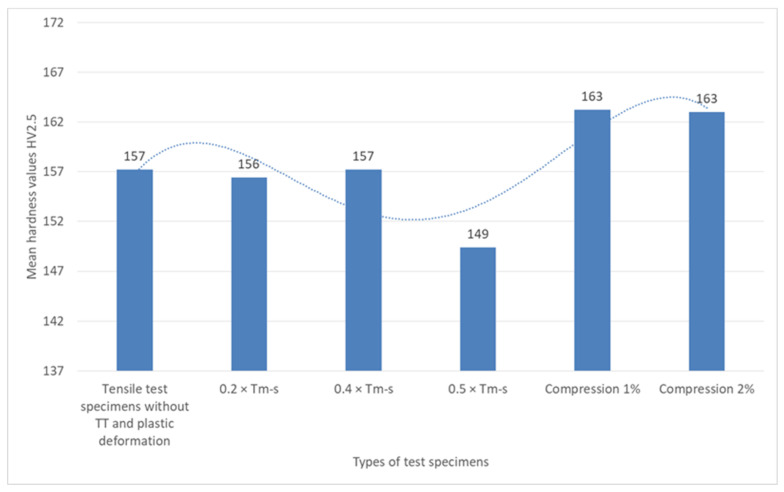
Mean material hardness values recorded in the Vickers hardness test with a load of 2.5 kgf for the six types of specimens tested.

Test data were graphically summarized as shown in Figure 6, Figure 7, Figure 8 and Figure 9 based on the results obtained from the experimental testing programs.

The variation in mechanical strength (σ_u_, expressed in MPa) for various specimens subjected to the test program described above is shown in the graph in Figure 6. Observations regarding the variation in σ_u_ are given below.

The highest value of mechanical strength, 633 MPa, is recorded for the specimens without heat treatment (TT) subjected to tensile test. This indicates that the material has a very good strength in its original condition, without any additional stress being applied.

In the case of the Specimens subjected to heat annealing without phase change at temperatures of 0.2 × T_m−s_ and 0.4 × T_m−s_, there is a reduction in mechanical strength, with σ_u_ dropping to 599 MPa in the former and to 596 MPa in the latter. This trend indicates that these types of treatments applied to the material under these conditions reduce the strength, possibly due to plastic deformation or reduction in the level of internal stresses.

The mechanical strength σ_u_ returns to higher values and reaches 623 MPa for specimens subjected to heat treatments without phase change at 0.5 × T_m−s_. This increase can be explained by a recrystallization effect or a structural rearrangement of the material.

It is observed that decreases slightly to 605 MPa at 1% compression and increases again to 611 MPa at 2% compression under conditions of plastic deformation by compression of the specimens (1% and 2%). The variations recorded may reflect a balance between negative (microcracking or fatigue) and positive (increased structural density) effects.

From the graph in Figure 7, which illustrates the variation of the yield strength σ_u_ (expressed in MPa) for the different specimens tested, it can be seen that for tensile specimens without heat treatment (TT), S_y_ has a value of 428 MPa, which is the yield strength of the material in its initial state, guaranteed by the manufacturer, without any other stresses.

For specimens subjected to non-phase change heat treatments at 0.2 × T_m−s_ and 0.4 × T_m−s_, S_y_ increases to 452 MPa, indicating a growth of elasticity, and at 0.4 × T_m−s_ the value slightly increases to 467 MPa, indicating a slight structural strengthening of the material after a certain temperature.

The highest value recorded for S_y_ reaches a peak of 486 MPa for specimens subjected to 0.5 × T_m−s_, suggesting a significant strengthening of the material due to structural deformation or rearranging the internal crystalline structure.

The specimens subjected to plastic deformation (1% and 2% compression) show a decrease to 464 MPa at S_y_, indicating a loss of elasticity at 1%, probably due to microcracking or structural relaxation. At 2% compression, the value decreases again to 451 MPa, suggesting a negative effect of the additional compression on the yield strength.

The graph in Figure 8 showing the values of relative strain variation (δ, expressed in percent) for the specimens under test show that the tensile specimens without heat treatment (TT) have a relative strain value of 14%, which is the behavior of the material in its initial state, guaranteed by the manufacturer.

The relative strain for specimens subjected to non-phase change heat treatments at 0.2 × T_m−s_ and 0.4 × T_m−s_ remains constant at 14%, indicating stability of material deformability at this stress level, suggesting that the material is not significantly affected in terms of ductility.

The relative strain peaks at 16%, indicating improvement in ductility, possibly related to internal structural reorganization or stress relief.

In specimens subjected to compression (1% and 2%), the relative strain decreases slightly to 15% at 1%, indicating a moderate decrease in the ductility of the material, and at 2% compression, the relative strain decreases to 12%, indicating a clear decrease in ductility, possibly due to the accumulation of internal defects, scaling or microcracking resulting from plastic deformation.

The analysis of the variation of the Vickers hardness at a load of 2.5 kgf (HV2.5) for the different sets of specimens, shown graphically in Figure 9, indicates a relatively small variation of the hardness with the state of the material.

The HV values are constant (approximately 157HV2.5) in the specimens without heat treatment and in the specimens with heat treatment without phase change at temperatures of 0.2 × T_m−s_ and 0.4 × T_m−s_, respectively.

The decrease of hardness in the treatment without phase change to 0.5 × T_s_ at 149HV2.5 indicates a possible increased influence of mechanical stress on the material, leading to a slight plastic deformation or a redistribution of internal stresses that may have an effect on the microstructure, which may signal the beginning of microstructure failure.

The hardness measurements of the compressive specimens (1% and 2%) yielded identical HV2.5 (163HV2.5) values. These values could be the result of the work hardening effect during the compression, which leads to an increase in the dislocation density and a higher local resistance to penetration. It has been demonstrated that deformation by a minimal percentage or a negligible relaxation of the microstructure at elevated levels of stress may be reflected in the hardness test value that remains constant.

Based on the analysis of these data, the next section discusses the results obtained in the experimental program of test specimens from the above-mentioned test specimens.

## 5. Discussion

Analyzing from the point of view of the material’s strength, it can be observed from the data analysis that both the application of heat treatments without phase change and plastic deformation for surface layer hardening result in a slight decrease in strength, by 10 to 37 MPa, which is not statistically significant.

As indicated by the mean values of yield strength noted S_y_, which are conventionally established by taking a parallel to the linear zone at the point on the deformation axis corresponding to a specific elongation ε = 0.002 (an elongation of 0.2% of L_0_) of the specimen, it is clear that for the heat treatment without a phase change at temperature 0.2 × T_m−s_ and for a 1% deformation of the specimens, there is a notable growth in elastic behavior, with values of 58 MPa and 36 MPa. Conversely, for the other treatments and 2% plastic deformation, there is an enhancement in elastic behavior, with values ranging between 13 and 29 MPa.

In examining the mean values for the relative ellipses, it is evident that specimens in their original, untreated state, as well as those subjected to heat treatment without phase change at temperatures of 0.2 × T_m−s_ and 0.4 × T_m−s_, do not exhibit any notable changes in this characteristic. The results show a decrease of 2% for specimens subjected to 2% compression and an improvement of 14% to 16% for those heat treated without phase change at 0.5 × T_m−s_ and subjected to 1% compression.

Regarding the mean values for relative elongation and hardness recorded after subjecting specimens to a Vickers test with a pressing force of 2.5 kN, the results yielded a similar outcome for the three specimen types, with the same value around to 157 HV2.5 being observed. Conversely, specimens subjected to heat treatment in the absence of a phase change at a temperature of 0.5 × T_m−s_ exhibited a notable decline in hardness, with an observed decrease of approximately 8 HV2.5. As expected, specimens that underwent plastic deformation with a 1% and 2% reduction in specimen height exhibited an increase in hardness values due to surface layer strain hardening. The observed increase was 6 HV2.5. It should be noted that at a higher degree of deformation, the strain hardening phenomenon would have resulted in a higher hardness value.

In applications where this material is used, such as in the construction of machine parts, the optimal result would be obtained by applying a phase change-free heat treatment at temperatures 0.5 × T_m−s_. This process would result in a mechanical strength value comparable to that of the untreated material, while improving the values of the breaking strength Sy and the relative elongation δ. Furthermore, the hardness value would remain relatively unchanged, which is not a significant concern in this particular context.

It is also of great importance for users of this material in various engineering applications to be able to select the appropriate heat treatment and plastic deformation processes, based on the results of the study, in order to guarantee that the desired mechanical properties of the designed parts are achieved, given the stresses to which they will be subjected.

The obtained results suggest that heat treatments without phase transformation induce microstructural changes that improve the strength and ductility of the material. A reorganization of the crystalline lattice can thus be observed which practically leads to variations in mechanical properties. These findings have significant implications for industrial applications, in particular in the field of metal construction and equipment made of S355J2+N steel.

## 6. Conclusions

A series of conclusions emerge from the study conducted by the authors and presented in this article. A first conclusion that emerges is that taking into account the average values of the mechanical characteristics derived from the mechanical tests, it can be demonstrated that the material exhibits the characteristics delineated in the standards SR EN-10025 and SR EN-10027 for steel S355J2+N, as evidenced by the results of this experimental program. This analysis provides essential information on the behavior of S355J2+N steel under various conditions, confirming its alignment with SR EN-10025 and SR EN-10027 specifications. While some treatments result in slight reductions in strength, these are not statistically significant. Some treatments improve elastic behavior, especially at 2% plastic deformation. This indicates improved material performance under certain conditions.

Another conclusion is that the study demonstrates that heat treatments devoid of phase transitions at specific temperatures (0.5 × T_m−s_) can enhance elongation and strength while maintaining minimal hardness, a property advantageous for applications that demand minimal hardness modification. Plastic deformation enhances hardness, particularly at elevated levels of deformation, a benefit that extends to applications necessitating surface hardening.

In terms of practical applications, such as the fabrication of machine components, the most effective approach entails subjecting the material to heat treatment at 0.5 × T_m−s_. This method ensures an optimal balance between mechanical strength, enhanced elongation, and improved tear strength performance, while maintaining minimal alterations in hardness.

In response to the research question that guided the development of this study, the answer is affirmative. The results obtained demonstrate that the application of heat treatments without phase change at the temperatures presented in the article, under the same conditions of chemical composition, led to the enhancement of the mechanical characteristics of the material, including ultimate material strength, yield strength, elongation, and hardness. It is proposed that future research directions include the execution of similar tests on different materials, the comparison of results with those obtained in this study, and the identification of a general relationship when applying these heat treatments without phase change and/or controlled plastic deformation.

Last but not least, the conclusions of this study underscore the significance of judicious selection of heat treatment and deformation processes to ensure that the mechanical properties of the material align with the demands of specific engineering applications.

## Figures and Tables

**Table 1:** Chemical composition of steel S355J2+N—steel strip 1. Source: Authors based on the manufacturer quality certificate.

Steel Strip	C	Si	Mn	*p*	S	Al	Ti	V	Cu	Ni	Cr	Mo	Nb	B	N2	CEV2
920,942	0.16	0.02	1.40	0.016	0.008	0.049	0.032	0.006	0.05	0.03	0.03	0.003	0.002	0.0001	0.0067	0.41

**Table 2:** Chemical composition of steel S355J2+N—steel strip 2. Source: Authors based on the manufacturer quality certificate.

Steel Strip	C	Si	Mn	*p*	S	Al	Ti	V	Cu	Ni	Cr	Mo	Nb	B	N2	CEV2
919,391	0.17	0.01	1.38	0.012	0.014	0.053	0.033	0.006	0.07	0.03	0.02	0.004	0.002	0.0001	0.0064	0.41

**Table 3:** Tensile test specimens.

Specimen No.:	Heat Treatment Type/ Plastic Deformation	L_i_ (mm)	h (mm)	L_f_ (mm)	L_m_ (mm)
1	No heat treatment or plastic deformation	150	10	173	171.6
2				170	
3				172	
4				171	
5				172	
6	0.2 × T_m−s_ 0.2 × 1812.15 K 90 °C			173	170.4
7				172	
8				171	
9				167	
10				169	
11	0.4 × T_m−s_ 0.4 × 1812.15 K 450 °C			172	171.2
12				171	
13				173	
14				170	
15				170	
16	0.5 × T_m−s_ 0.5 × 1812.15 K 635 °C			172	173.4
17				173	
18				171	
19				170	
20				172	
26	Compression stressed—1%		9.9	171	171.8
23			9.86	170	
24			9.87	171	
27			9.84	174	
29			9.92	173	
21	Compression stressed—2%		9.83	169	168.6
22			9.68	167	
25			9.81	170	
28			9.83	170	
30			9.82	167	

**Table 4:** Vickers hardness test specimens with a force of 2.5 kgf.

Specimen No.:	Heat Treatment Type/ Plastic Deformation	h (mm)
1	No heat treatment or plastic deformation	5
2		
3		
4		
5		
6	0.2 × T_m−s_ 0.2 × 1812.15 K 90 °C	
7		
8		
9		
10		
11	0.4 × T_m−s_ 0.4 × 1812.15 K 450 °C	
12		
13		
14		
15		
16	0.5 × T_m−s_ 0.5 × 1812.15 K 635 °C	
17		
18		
19		
20		
21	Compression stressed—1%	4.87
22		4.89
23		4.88
24		4.94
25		4.88
26	Compression stressed—2%	4.8
27		4.83
28		4.82
29		4.84
30		4.85

**Table 5:** Tensile test results for the tested specimens.

Specimen No.:	L_i_ (mm)	h (mm)	L_f_ (mm)	σ_u_ [MPa]	S_y_ [MPa]	δ [%]
No heat treatment or plastic deformation specimens						
1	150	10	173	605	405	15.33
2			170	625	423	13.33
3			172	648	434	14.66
4			171	637	440	14
5			172	649	438	14.66
Mean value				632.8	428	14.396
Standard deviation				16.400	12.962	0.679
Heat-treated test specimens without phase transformation at 0.2 × T_m−s_						
6	150	10	173	520	410	15.33
7			172	611	475	14.66
8			171	550	400	14
9			167	665	485	11.33
10			169	650	490	12.66
Mean value				599.2	452	13.596
Standard deviation				56.112	38.807	1.436
Heat-treated test specimens without phase transformation at 0.4 × T_m−s_						
11	150	10	172	560	445	14.66
12			171	570	460	14
13			173	582	470	15.33
14			170	595	478	13.33
15			170	673	480	13.33
Mean value				596	467	14.13
Standard deviation				40.244	12.893	0.777
Heat-treated test specimens without phase transformation at 0.5 × T_m−s_						
16	150	10	174	665	500	16
17			173	650	495	15.33
18			174	621	502	16
19			174	568	460	16
20			172	610	473	14.66
Mean value				622,8	486	15.598
Standard deviation				33.737	16.636	0.536
Non-heat-treated specimens under the application of a compressive load 1%						
21	150	9.9	171	650	490	14
22		9.86	170	642	487	13.33
23		9.87	171	530	446	14
24		9.84	174	590	440	16
25		9.92	173	615	455	15.33
Mean value				605.4	464	14.532
Standard deviation				43.228	20.906	0.980
Non-heat-treated specimens under the application of a compressive load 2%						
26	150	9.83	169	652	477	12.66
27		9.68	167	565	432	11.33
28		9.81	170	590	419	13.33
29		9.83	170	660	500	13.33
30		9.82	167	600	428	11.33
Mean value				613.4	451	12.396
Standard deviation				36.691	31.439	0.904

**Table 6:** Vickers 2.5 kgf hardness test results. Source: Authors based on the content of the research results.

Specimen No.:	h (mm)	Hardness (HV 2.5)
No heat treatment or plastic deformation specimens		
1	5	157
2		156
3		158
4		157
5		158
Mean value		157.2
Standard deviation		0.748
Heat-treated test specimens without phase transformation at 0.2 × T_m−s_		
6	5	155
7		157
8		158
9		159
10		154
Mean value		156.6
Standard deviation		1.854
Heat-treated test specimens without phase transformation at 0.4 × T_m−s_		
11	5	156
12		157
13		156
14		159
15		158
Mean value		157.2
Standard deviation		1.166
Heat-treated test specimens without phase transformation at 0.5 × T_m−s_		
16	5	149
17		150
18		151
19		148
20		149
Mean value		149.4
Standard deviation		1.019
Non-heat-treated specimens under the application of a compressive load 1%		
21	4.87	164
22	4.89	163
23	4.88	162
24	4.94	163
25	4.88	164
Mean value		163.2
Standard deviation		0.748
Non-heat-treated specimens under the application of a compressive load 2%		
26	4.8	160
27	4.83	161
28	4.82	162
29	4.84	158
30	4.85	159
Mean value		160.0
Standard deviation		1.414

## Data Availability

The original contributions presented in this study are included in the article. Further inquiries can be directed to the corresponding authors.
